# Channel-Unet: A Spatial Channel-Wise Convolutional Neural Network for Liver and Tumors Segmentation

**DOI:** 10.3389/fgene.2019.01110

**Published:** 2019-11-26

**Authors:** Yilong Chen, Kai Wang, Xiangyun Liao, Yinling Qian, Qiong Wang, Zhiyong Yuan, Pheng-Ann Heng

**Affiliations:** ^1^Shenzhen Institutes of Advanced Technology, Chinese Academy of Sciences, Shenzhen, China; ^2^AI Research Center, Peng Cheng Laboratory, Shenzhen, China; ^3^CAS Key Laboratory of Human-Machine Intelligence-Synergy Systems, Shenzhen Institutes of Advanced Technology, Shenzhen, China; ^4^Guangdong Provincial Key Laboratory of Computer Vision and Virtual Reality Technology, Shenzhen Institutes of Advanced Technology, Shenzhen, China; ^5^School of Computer Science, Wuhan University, Wuhan, China; ^6^T Stone Robotics Institute and Department of Computer Science and Engineering, The Chinese University of Hong Kong, Shatin, Hong Kong

**Keywords:** liver and tumors segmentation, computed tomography, deep learning, spatial channel-wise convolution, Channel-UNet

## Abstract

It is a challenge to automatically and accurately segment the liver and tumors in computed tomography (CT) images, as the problem of over-segmentation or under-segmentation often appears when the Hounsfield unit (Hu) of liver and tumors is close to the Hu of other tissues or background. In this paper, we propose the spatial channel-wise convolution, a convolutional operation along the direction of the channel of feature maps, to extract mapping relationship of spatial information between pixels, which facilitates learning the mapping relationship between pixels in the feature maps and distinguishing the tumors from the liver tissue. In addition, we put forward an iterative extending learning strategy, which optimizes the mapping relationship of spatial information between pixels at different scales and enables spatial channel-wise convolution to map the spatial information between pixels in high-level feature maps. Finally, we propose an end-to-end convolutional neural network called Channel-UNet, which takes UNet as the main structure of the network and adds spatial channel-wise convolution in each up-sampling and down-sampling module. The network can converge the optimized mapping relationship of spatial information between pixels extracted by spatial channel-wise convolution and information extracted by feature maps and realizes multi-scale information fusion. The proposed ChannelUNet is validated by the segmentation task on the 3Dircadb dataset. The Dice values of liver and tumors segmentation were 0.984 and 0.940, which is slightly superior to current best performance. Besides, compared with the current best method, the number of parameters of our method reduces by 25.7%, and the training time of our method reduces by 33.3%. The experimental results demonstrate the efficiency and high accuracy of Channel-UNet in liver and tumors segmentation in CT images.

## Introduction

Automatic liver and tumors segmentation in medical images has great significance in qualitative analysis of hepatic carcinoma, which can facilitate surgeons to diagnose disease and plan the patient-specific surgery (Vorontsov et al., 2019). Computed tomography (CT) is the main modality to diagnose hepatic carcinoma, while the CT images are characterized by collections of interrelated objects with uneven gray levels and gray similarities, linked together into complex graphs and structures. It is a challenging task to achieve accurate and automatic segmentation of liver and tumors in CT images, as the problem of over-segmentation or under-segmentation often appears when the Hounsfield unit (Hu) of tumors is close to the liver tissue, especially for 3D CT images due to large data scale and computation (Moghbel et al., 2018; Qin et al., 2018; Liu et al., 2019a).

**Figure 1:**
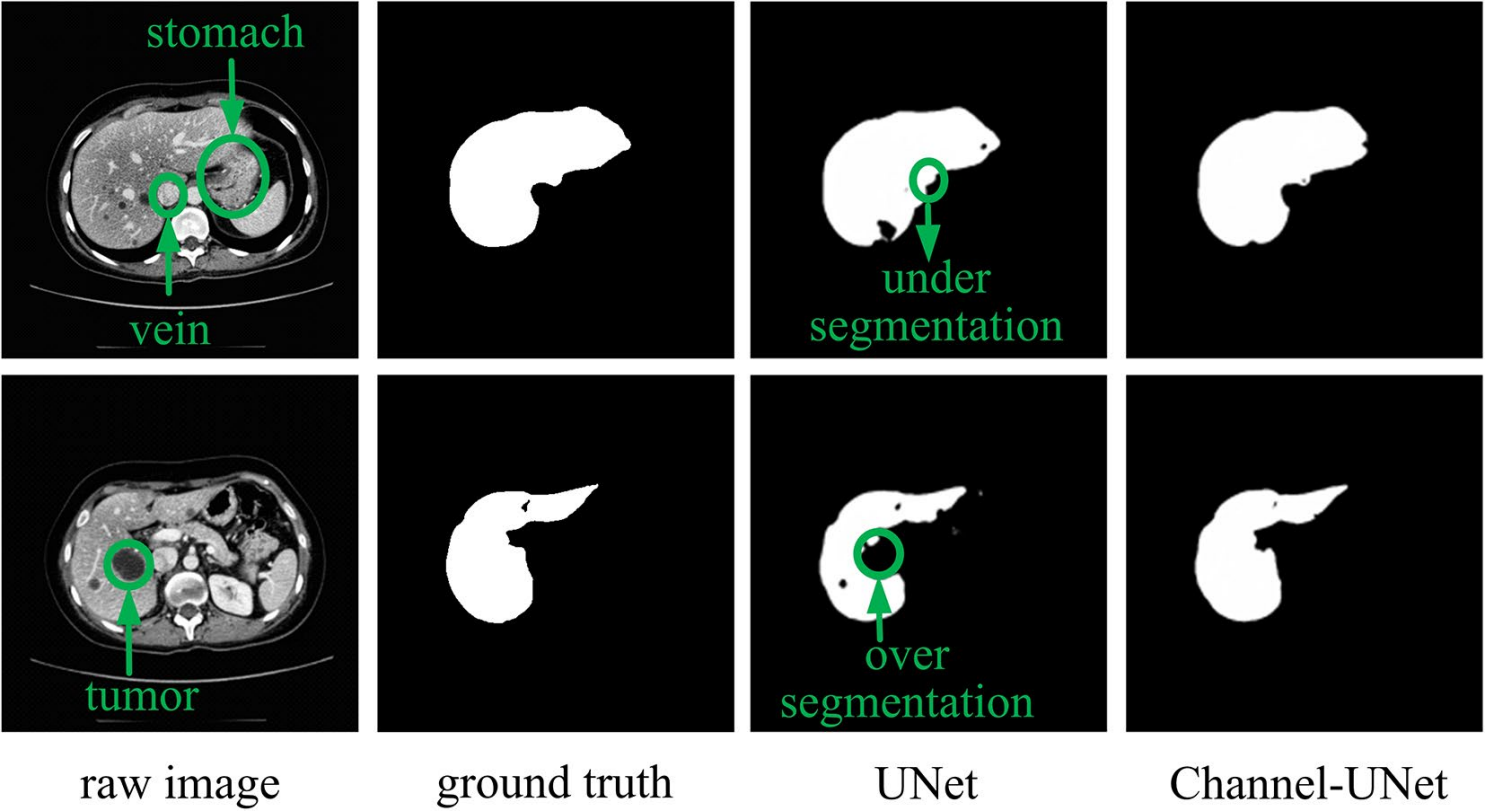
Example of over-segmentation and under-segmentation in liver segmentation.

To accurately segment liver and tumors in CT images, numerous segmentation methods have been proposed, including intensity threshold, region growth, and deformation model. In recent years, the development of convolutional neural networks, especially fully convolutional neural networks (FCN) (Long et al., 2015), has made great achievements in the field of semantics segmentation, such as the methods in (Lee et al., 2019; Zhou et al., 2019; Liu et al., 2019b). However, in dealing with the problem of image segmentation, convolutional neural networks may exaggerate the difference between similar objects (inter-class distinction) or the similarity between different kinds of objects (intra-class consistency) (Yu et al., 2018). This problem is especially serious in the field of medical image segmentation due to the similarity of physical characteristics of various human tissues that the Hu of various tissues is overlap with each other. For example, in liver segmentation, the Hu of the liver tissue is very close to that of the adjacent stomach (as shown in first row of **Figure 1**), and the Hu value of the tumor is close to the background (as shown in second row of **Figure 1**). This ultimately makes it difficult for the neural network to distinguish the boundary between the target tissues (liver, tumors) and other similar soft tissues or background.

To tackle with the above difficulty, we need to locate the target tissue and extract the features of similar objects, and solve the problems of "inter-class difference" and "intra-class consistency" (Liu et al., 2015). To solve the problem of "inter-class difference," (Liu et al., 2015) introduced the global context information and combined the original feature map with the pooled images to fully utilize the global information. Similar studies (He et al., 2015; Chen et al., 2017; Li et al., 2018a) also used the idea of global context feature to select and combine low- and high-level features. To solve the problem of "intra-class consistency," researchers mainly added the edge detection network to the original segmentation network, thus the whole network can realize both segmentation and edge detection (Chen et al., 2016a; Cui et al., 2018) to distinguish the boundaries between similar classes.

However, the above methods lack the pixel-level mapping relationship of spatial information between pixels, which would result in wrongly detecting the similar tissues as the target tissue, as well as failing to distinguish various sub-domains with difference of the target tissue. To address this problem, we consider to extract the mapping relationship of spatial information between pixels from different convolutional layers of UNet (Ronneberger et al., 2015). The core idea is that, in convolutional neural network, although the feature maps obtained by convolutional operation retain spatial information, there is no extracted mapping relationship of spatial information between pixels, making it difficult to perform iterative spatial mapping optimization by loss function. Therefore, we consider extracting the mapping relationship of spatial information between pixels for identifying the differences of similar tissues and commonalities of different tissues. In this paper, we propose spatial channel-wise convolution, a 1×1 convolutional operation along the channel of feature map to extract the mapping relationship of spatial information between pixels in the feature maps. Then, we put forward an iterative extending learning strategy, which optimizes the spatial information at multiple scales. Finally, we propose the Channel-UNet, which can effectively converge the optimized spatial information extracted by spatial channel-wise convolution and the existing information extracted by UNet in the feature maps. The third and the fourth column of **Figure 1** show a comparison of liver segmentation results with UNet (Ronneberger et al., 2015) and the proposed Channel-UNet, the experimental results demonstrate that the proposed method can effectively alleviate the problems of over-segmentation and under-segmentation.

The contributions of this paper are as follows:

- We propose the spatial channel-wise convolution, a convolutional operation along the direction of the channel of the feature maps, which facilitates the neural network to learn the mapping relationship between pixels in feature maps and to distinguish liver and tumors from other similar tissues or background.
- We put forward an iterative extending learning strategy. The proposed learning strategy extends the receptive field of single spatial channel-wise convolution layer by layer, and obtains spatial channel-wise convolution at multiple scales by max-pooling and transpose convolution. Thus, the receptive field of spatial channel-wise convolution at multiple scales is increased and the mapping relationship of spatial information between pixels can be effectively optimized iteratively by back propagation.
- We propose the Channel-UNet, which takes UNet as the main framework. Each module in the UNet consists of the convolution layer and the spatial channel-wise convolution layer. By concatenating spatial channel-wise convolution and feature map along the direction of the channel of the feature maps, the Channel-UNet can realize multi-scale information fusion and effectively suppress the over-segmentation and under-segmentation problems in the process of segmentation.

### Related Work

Convolutional neural networks have been applied to various medical image segmentation tasks. However, medical images contains various soft tissues with complex structures, we need to distinguish the target tissue from various soft tissues. Current research mainly focuses on adding multi-scale image information and optimizing the extracted image information by using attention-aware methods to achieve accurate segmentation.

### Multi-Scale Context-Based Methods

In image segmentation, the convolution network can successfully determine the coarse boundary of the target by extracting the abstract features of the image. Generally, the deeper the network is, the more complex and abstract the information can be fitted with the increase of parameters and receptive field of convolution kernel. This success can be partly attributed to the inherent invariance of local image transformation in the convolution network, which enables the network to learn higher-level feature. This invariance is helpful for higher-level tasks, but it may hinder low-level tasks. For example, in image segmentation, both the high-level abstract information and low-level pixel information are required for accurate segmentation. While with the increasing of the numbers of neural network layers, the information at low-levels is prevented from being transmitted to the output. To solve this problem, researchers propose two solutions. The first solution is to use low-level feature information to assist high-level feature to restore image details. Specifically, up-sampling operations are used to encode the information and recover the missing target details due to the down-sampling process by connecting the encoding network with the decoding network. For example, FCN (Long et al., 2015) and UNet (Ronneberger et al., 2015) use transposed convolution for up-sampling, and then connect the shallow convolutional layers with the deep convolutional layers by jump connection, so that the network can make full use of the shallow information of the convolutional neural network. However, these methods cannot retrieve the spatial information loss in the process of pooling and convolution. Although the dilated convolution proposed in (He et al., 2015) can avoid this problem, it consumes a lot of computing resources. The second solution is to use the semantic information in the middle layer of the network to generate high-resolution prediction results, thus reducing the information loss in the encoding stage due to the addition of pooling layer, such as multi-path refinement network (Lin et al., 2017) and GCN network (Peng et al., 2017). By using ResNet (He et al., 2016), the feature maps output in four different down-sampling stages are input into refined net module to generate feature maps with both rough high-level semantic features and fine low-level semantic features.

### Attention-Aware Methods

The convolutional layer contains the spatial information of each pixel in the feature maps. Therefore, many researchers try to optimize the spatial information of convolutional layers. According to the location of the convolutional layers in the network structure, the optimization of spatial information extracted from feature maps can be divided into encoding, decoding and output stages. In the stage of network encoding and decoding, attention mechanism is often applied to optimize the extracted spatial information of feature maps (Chen et al., 2016b; Firat et al., 2016). The basic idea is to generate a mask between 0 and 1 by transformation, and then multiply it with the original feature maps. In this way, the region of interest remains unchanged, the rest of the image becomes zero. However, the direct application of attention mechanism would result in the loss of useful information when wrong judgment of the region of interest occurs. To solve this problem, Wang et al. (2017) and Zhu et al. (2017) proposed to add soft mask branch to the original convolutional layers to enhance the area activated by the convolution kernel while retaining the original decoding network. Thus, even if the attention mechanism fails, the original image information still can be corrected in the later training process. In addition to applying attention mechanism to decoding stage, (Hong et al., 2016) applies attention mechanism to the encoding stage, and their method multiplies the feature maps with the feature maps after applying attention mechanism. As a result, the graph after attention mechanism and the feature maps before attention mechanism can undergo a reversible matrix transformation to ensure that the useful information will be retained. In the image output stage, conditional random field is usually introduced to post-process the segmented images (Christ et al., 2016), it can facilitate the smoothing the edge of the segmentation.

## Method

In this paper, we consider extracting and optimizing the mapping relationship of spatial information between pixel in convolution layers to solve the problems of over-segmentation and under-segmentation. We propose the spatial channel-wise convolution, iterative extending learning strategy, and Channel-UNet framework, which can converge the optimized mapping relationship of spatial information extracted by spatial channel-wise convolution and the existing information extracted by UNet in the feature maps, thus achieving accurate liver and tumors segmentation in CT images.

### Network Architecture

**Figure 2:**
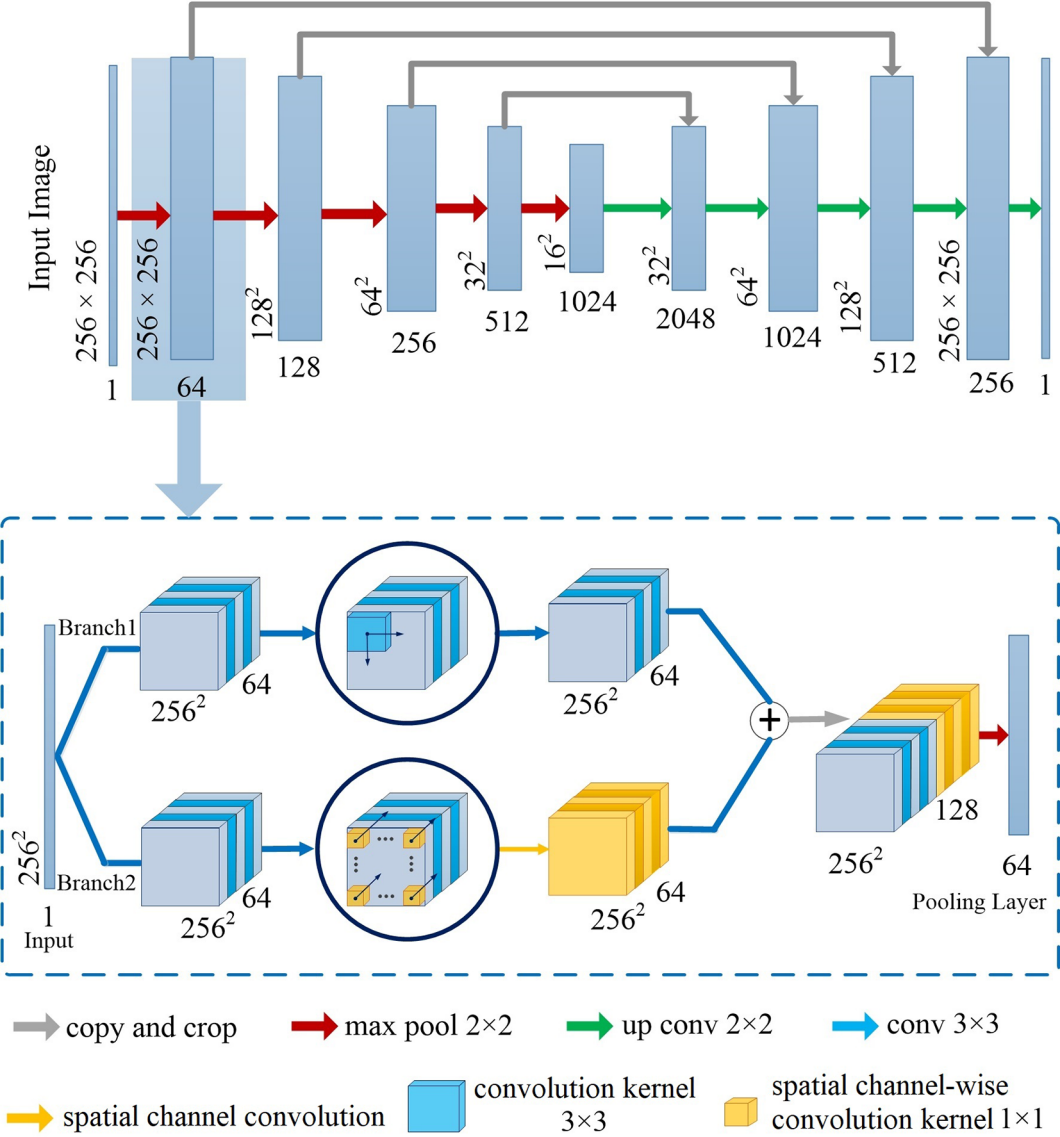
Network architecture of Channel-UNet.

**Figure 2** illustrates the network architecture of Channel-UNet. To be more specific, we adopt UNet (Ronneberger et al., 2015) as the backbone structure of the proposed Channel-Net, which forms a symmetrical structure, as shown in the top of **Figure 2**. The encoding network extracts the feature information, while the decoding network reconstruct the feature information. The encoding and decoding networks are connected by jump connection, thus the network can retain shallow information. Each sub-module of the backbone network is connected in series by max pooling layer and transposed convolution, as shown in the bottom of **Figure 2**. Each sub-module consists of two branch channels. Branch 1 is composed of multiple convolutional layers in series. Branch 2 is composed of multiple convolution layers and a spatial channel-wise convolution layer in series, which would extend the receptive field of the spatial channel-wise convolutional layers. The two branches are eventually concatenated. The purpose of this step is to preserve the information of the original convolution layers, such as pixel information and shape information, when extracting mapping relationship of spatial information between pixels by spatial channel-wise convolution. The Channel-UNet adopts the iterative extending learning strategy to train the network, the mapping relationship of spatial information between pixels can be effectively optimized iteratively by back propagation. Finally, the optimized mapping relationship of spatial information extracted by spatial channel-wise convolution and the feature information extracted by convolution are converged by max-pooling layers. In practice, batch normalization layer (Normalization, 2015) and *L2* regularization are added between convolution layers to effectively alleviate over-fitting.

### Spatial Channel-Wise Convolution

The lacking of mapping relationship of spatial information makes it difficult to perform iterative spatial mapping optimization, which would result in the problems of over-segmentation and under-segmentation. In this paper, we propose the spatial channel-wise convolution, a convolutional operation along the direction of the channel of feature maps, which extracts the mapping relationship of spatial information in convolutional layers and facilitates distinguishing liver and tumors from other similar tissues or background.

**Figure 3:**
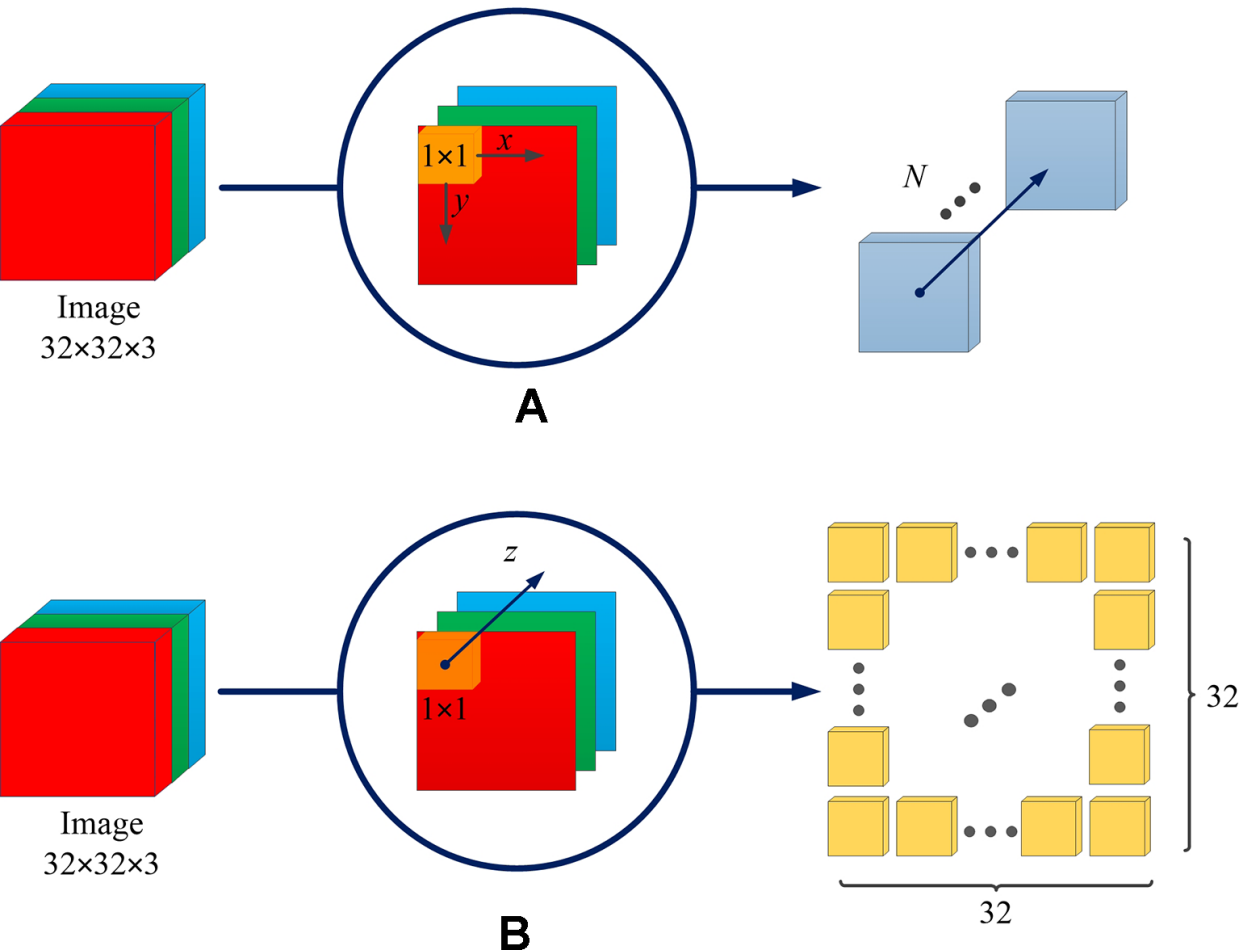
The difference between convolution and spatial channel-wise convolution. **(A)** Convolution: the convolution kernel slides on the (*x*, *y*)-plane, and the resulting feature map is concatenated along the *z*-axis direction, which is the direction of the channel of the feature maps. **(B)** Spatial channel-wise convolution: the convolution kernel slides in the *z*-axis direction, and the resulting feature maps are arranged in the (*x*, *y*)-plane. The arrangement rules are the same as those of the input feature maps.

The difference between the spatial channel-wise convolution and the convolution is the sliding direction of the convolutional kernel. Specifically, in the traditional convolution, the convolution kernel slides on the (*x*, *y*)-plane of the image, while in the spatial channel-wise convolution, the spatial channel-wise convolution kernel slides on the channel *z*-axis of the image. **Figure 3** demonstrates the difference between convolution and spatial channel-wise convolution with three input images (32 × 32). Traditional convolution use 1 × 1× *N* convolution kernels, where *N* represents the number of convolutional kernels whose value is equal to the number of output images. In order to calculate its output, the 1 × 1 convolution kernel is multiplied by the corresponding pixel value in each input image, respectively, and then the sum is obtained. Secondly, the 1 × 1 convolutional kernel slides in (*x*, *y*)-plane. Finally, the *N* convolution results are superimposed along the *z*-axis direction, which is the channel of feature maps, as shown in **Figure 3A**. When the step size is 1 and the filled convolution kernel is not included, the convolutional operation, using the 1×1 convolution kernel, is equivalent to the multiplication of matrices, as shown in Equation 1.

(1) $$a^{l}=\delta (Z^{l})=\delta (\sum_{k=1}^{N}a_{i,j,k}^{l-1}w_{k}^{l}+b^{l})$$

where *l* is the number of layers, *b* is the offset, δ is the activation function, *Z*_(_ *_i_*_,_ *_j_*_)_ is the pixel value of the corresponding feature map, *N* is the number of channels of the feature map, *w* is the weight coefficient.

For spatial channel-wise convolution, as shown in **Figure 3B**, we use 1 × 1 × 32^2^ convolutional kernels to calculate spatial channel-wise convolution with three input images (32 × 32). The 1 × 1 convolutional kernel is placed at the top left corner, and then multiplied by the pixel value of the first input image (red image). Then the convolutional kernel slides along the *z*-axis, multiplying the corresponding pixel value of the second (green) and third (blue) input images. Finally, the results of *k* convolution kernels are arranged in the (*x*, *y*)-plane according to the original position of spatial channel-wise convolution kernels. The previous steps can be expressed in Equation 2.

(2) $$a^{l}=\delta (z^{l}{\;}_{\left(i,j\right)})=\delta (a_{k}^{l-1}⊙w_{\left(i,j\right)}^{l}+b^{l})$$

where *l* is the number of layers, *b* is the offset. δ is the activation function, *z*_(_ *_i_*_,_ *_j_*_)_is the pixel value of the corresponding feature map, *k* is the number of channels of the feature map, *w* is the weight. coefficient, denotes Hadamard product.

### Iterative Extending Learning Strategy

By using spatial channel-wise convolution, we extract the mapping relationship of spatial information between pixels, then we need to optimize the extracted mapping relationship of spatial information for accurate segmentation of liver and tumors. In this paper, we put forward an iterative extending learning strategy to optimize the mapping relationship of spatial information between pixels at different scales and enables spatial channel-wise convolution to map the spatial information between pixels in high-level feature maps. The receptive field of spatial channel-wise convolution at multiple scales is iteratively extended and the mapping relationship of spatial information between pixels can be efficiently optimized iteratively by back propagation.

**Figure 4:**
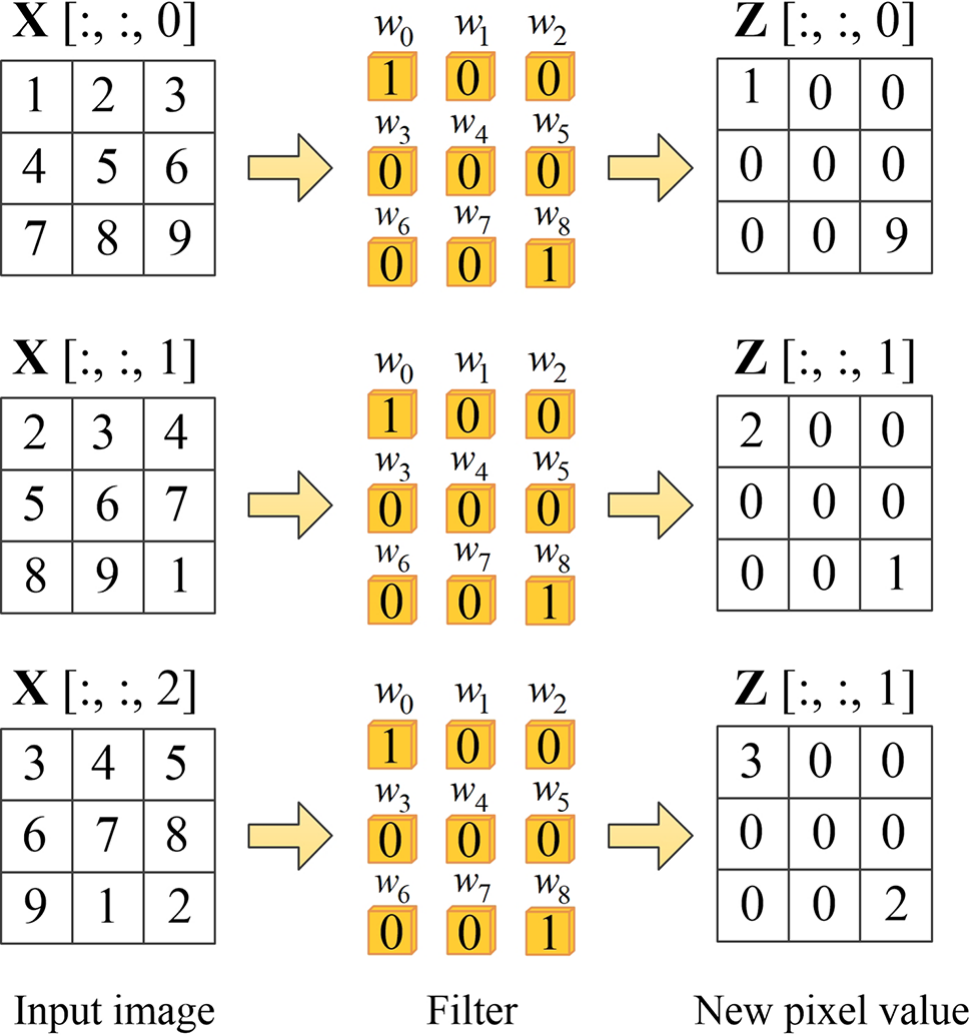
Example of learning mapping relationship between two pixels. The first column on the left represents the pixel values on three channels of the input volume, the second column represents spatial channel-wise convolution, and the third column is the output of each channel after the spatial channel-wise convolution.

To be more specific, when the input images pass through the spatial channel-wise convolution layer, the pixels in the image are activated by different convolution kernels and the mapping relationship is established between the pixels in the input images. For convolution layer (*l* −1), the input feature of layer (*l* −1) is mapped to **X**. We denote **Z** as the output feature of layer *l*, which can be obtained by spatial channel-wise convolution calculation, as shown in Equation 3. **Figure 4** demonstrates an example of learning mapping relationship between two pixels. When nine different 1 × 1 convolution kernels are applied to a 3 × 3 image, the pixel values of both upper left and lower right corners are 1, while the pixel values of the other locations are 0, thus we can learn the mapping relationship between the upper left and lower right pixels.

(3) $$Z^{l}=W^{l}⊙X^{l-1}+b^{l}$$

where **X** is the input image, **W** is the convolution kernel variable, **b** is the offset, *l* is the index of layers number, and **Z** is the output value.

According to Equation 3, the partial derivative of loss function with respect to **W** of layer *l* is shown in Equation 4. Similarly, the partial derivative of the loss function with respect to the offset **b** of layer *l* is shown in Equation 5.

(4) $$\frac{\partial ℒ(y,\hat{y})}{\partial W^{\left(l\right)}}=\frac{\partial ℒ(y,\hat{y})}{\partial Z^{\left(l\right)}}⊙X^{\left(l-1\right)}$$

(5) $$\frac{\partial ℒ(y,\hat{y})}{\partial b^{\left(l\right)}}=\frac{\partial ℒ(y,\hat{y})}{\partial Z^{\left(l\right)}}$$

where *L* is the loss function, y is the ground truth, ŷ is the predicted value.

**Figure 5:**
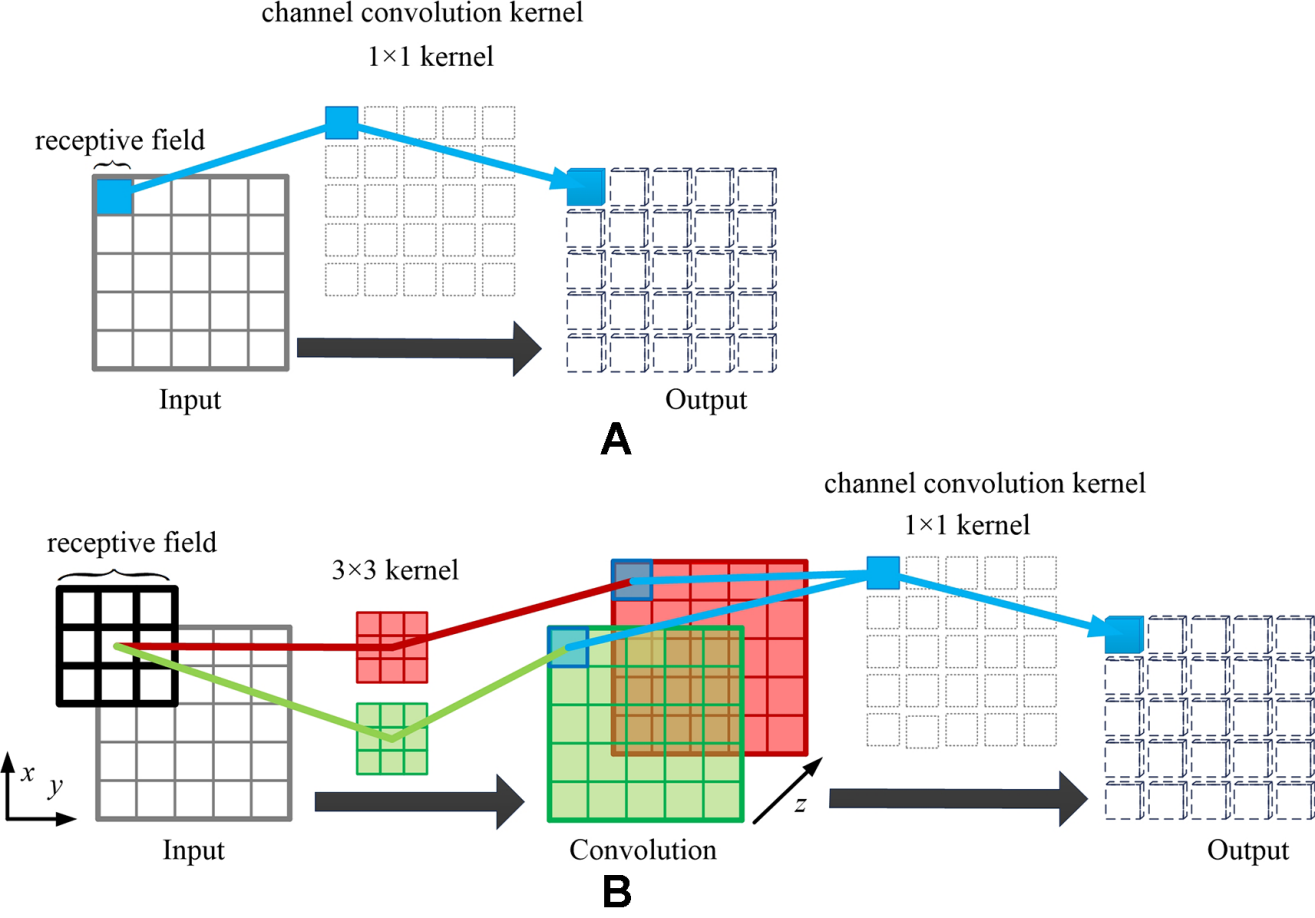
Example of extending receptive field of spatial channel-wise convolution. Taking the blue pixel as example, when only using the spatial channel-wise convolution, as shown in **(A)**, the effective receptive field of the red pixel in the output feature map is 1, which is the corresponding blue pixel in the input feature map. When adding the traditional convolution layer before spatial channel-wise convolution (convolution kernel size = 3, padding = 1, stride = 1), as shown in **(B)**, the effective receptive field of the blue pixel in the output feature map is 3, which is the same size of the convolution kernel in the input feature map.

It can be observed in Equations 4 and 5 that the spatial relationship between the pixels can be iteratively optimized by back propagation after spatial channel-wise convolution. However, the receptive field of 1 × 1 kernel convolution is limited, making it difficult to learn the mapping relationship of high-level spatial features in the images. Fortunately, we can stack convolution layers to extend the receptive field of spatial channel-wise convolution. According to the characteristics of the receptive field in convolutional neural network, with the increase of the number of convolution layers, the receptive field of each feature point in the feature map corresponding to the original image increases. Therefore, in order to map the relationship between different depth features, we consider adding convolutional layers to the spatial channel-wise convolution layers, thus the relationship between deep image features can be mapped by spatial channel-wise convolution. As shown in **Figure 5**, a convolution layer with 3 × 3 kernel and step size of 1 is added before spatial channel-wise convolution, the receptive field of spatial channel-wise convolution will be equal to that of convolution layer. In this way, we can represent the mapping relationship between pixels in different receptive field with only a 1 × 1 spatial channel-wise convolutional kernel. As a result, 1 × 1 spatial channel-wise convolutional kernel can learn the high-level mapping relationship of spatial information between pixels by iterative extending learning strategy.

## Experimental Results

In order to evaluate the performance of Channel-UNet, the experiment was carried out on the open dataset of 3Dircadb^1^. The experiment consists of two parts: in the first part, we conduct the liver segmentation experiment to test performance of Channel-UNet under different receptive field. In the second part, we adopt the method of ablation research to validate the effectiveness of spatial channel-wise convolution. The 3Dircadb data set contains 20 abdominal CT images. It also indicates the major drawbacks during liver segmentation due to the existing of neighboring organs. Therefore, it is convenient to test the performance of the proposed network in dealing with different drawbacks.

### Implementation Detail

**Figure 6:**
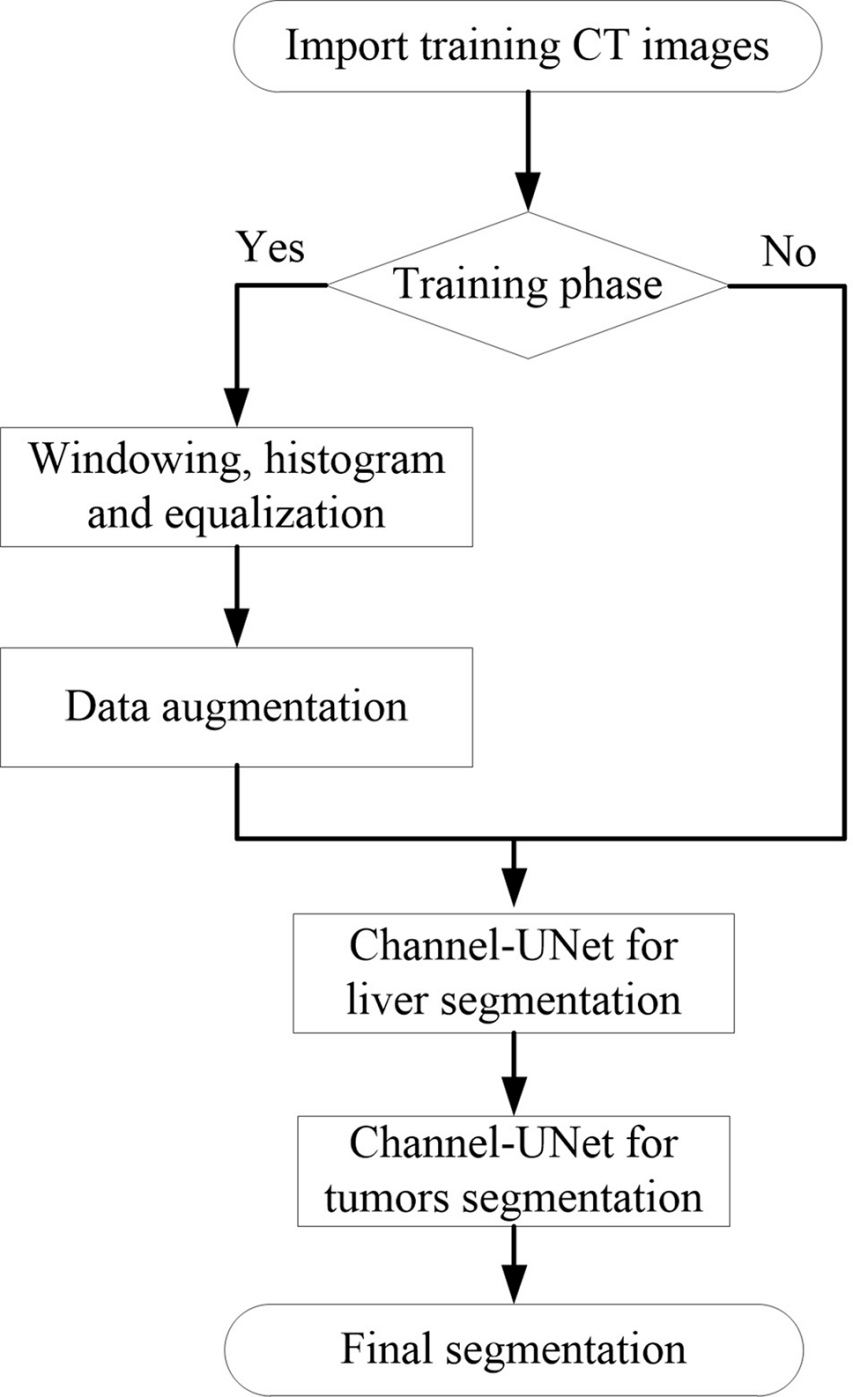
The illustration of the pipeline for Channel-UNet.

The model was implemented using Keras package (Chollet et al., 2015). The experiments were implemented on a platform with two GTX 1080 GPUs (8 GB display memory). **Figure 6** illustrates the entire training and testing process.

Inspired by (Wang et al., 2017), before training, the medical image preprocessing was carried out in a slice-wise fashion. First, the intensity values of all scanned images are truncated to (−200, 250) Hu to enhance the contrast between the liver and the surrounding organs and tissues, in order to remove irrelevant organs and tissues. Second, we crop the 512×512 scale data to 256×256 so as to reduce the amount of parametric quantity and increase the proportion of foreground regions. Third, because the training data provided by the dataset is small, horizontal flip and perspective transformation are used to enhance the training set and effectively alleviate the over-fitting of the model. Finally, since the original slice data are too small in the target area at the beginning and the end, five slices will be discarded at the end of the training. The training process is divided into three stages. First, the liver is segmented by Channel-UNet. Then, the tumors are segmented by Channel-UNet. Finally, the tumors are segmented by cascaded Channel-UNet. We train the network using mini-batch adaptive moment estimation (Adam) (Kingma and Ba, 2014) with batch size 2, learning rate 0.00001. Loss function is set to Dice loss function. The number of training iterations is set to 100, and the training is stopped when over fitting occurs. In this paper, when generalization errors increase within *N* (here we set *N* = 8) continuous epochs, the over fitting occurs and we stop training the network.

In the test, 10% of the training data were randomly extracted as the verification set, and the results were verified by 10-fold cross-validation. For measuring the performance of our proposed network, five metrics are used to measure the accuracy of segmentation results including the Dice, which refers to the same measurement as Dice per case in 3Dircadb dataset, average symmetric surface distance (ASD), volumetric overlap error (VOE), relative volume difference (RVD), and root mean square symmetric surface distance (RMSD). For the last four evaluation metrics, smaller values indicate better segmentation results.

### The Effect of Extending Receptive Field on Spatial Channel-Wise Convolution

**Figure 7:**
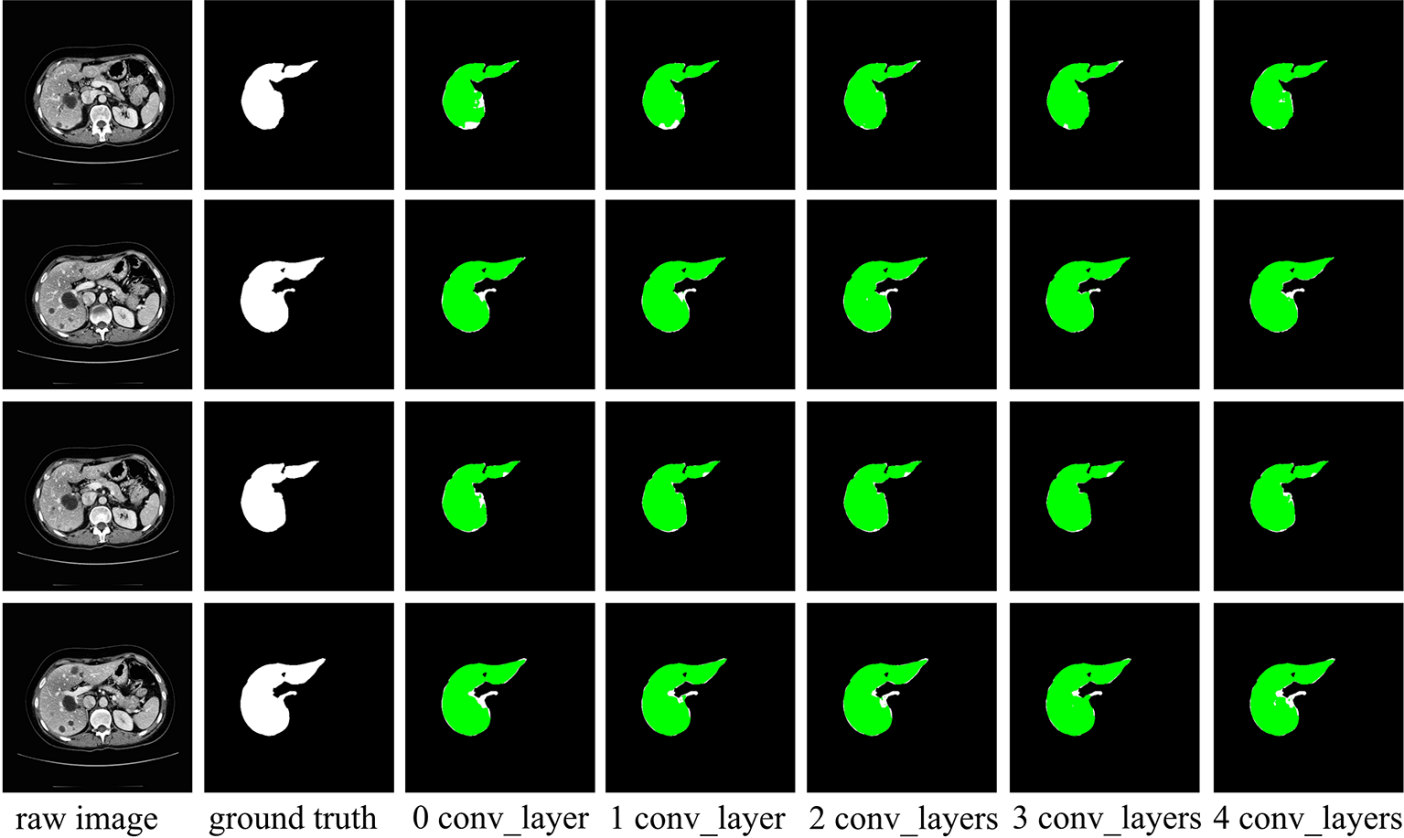
Examples of liver segmentation from different receptive field. The first column is the original image, the second column is the true liver segmentation, and the columns 3 to 7 show the effect of different number of convolutional layers in front of spatial channel-wise convolution layer. The green region denotes the liver.

According to the analysis in *Iterative Extending Learning Strategy*, the receptive field of spatial channel-wise convolution can be improved by adding convolutional layer before spatial channel-wise convolution layer. In order to further test its effect in practical application, in this section we test the effect of spatial channel-wise convolution on liver segmentation under different receptive field by changing the number of convolutional layers in front of spatial channel-wise convolution layer. The network structure is based on the network introduced in *Network Architecture*. The number of convolutional layers in branch 2 is {0,1,2,3,4} in the experiment. **Table 1** lists the dice values of the segmented liver. The experimental results are shown in **Figure 7**. It can observed in the experimental results that with the increase of the number of convolutional layers stacked in front of spatial channel-wise convolution layer, the Dice value increases first and then decreases. Through the experiment, we found out that when the convolutional layer number is 3, the Dice value is the highest.

**Table 1:** The performance of Channel-UNet with different number of convolutional layers.

Number of convolution layers	Dice (%)
0	92.6
1	94.2
2	97.4
3	98.4
4	96.3

### Ablation Study for Channel-Unet

**Figure 8:**
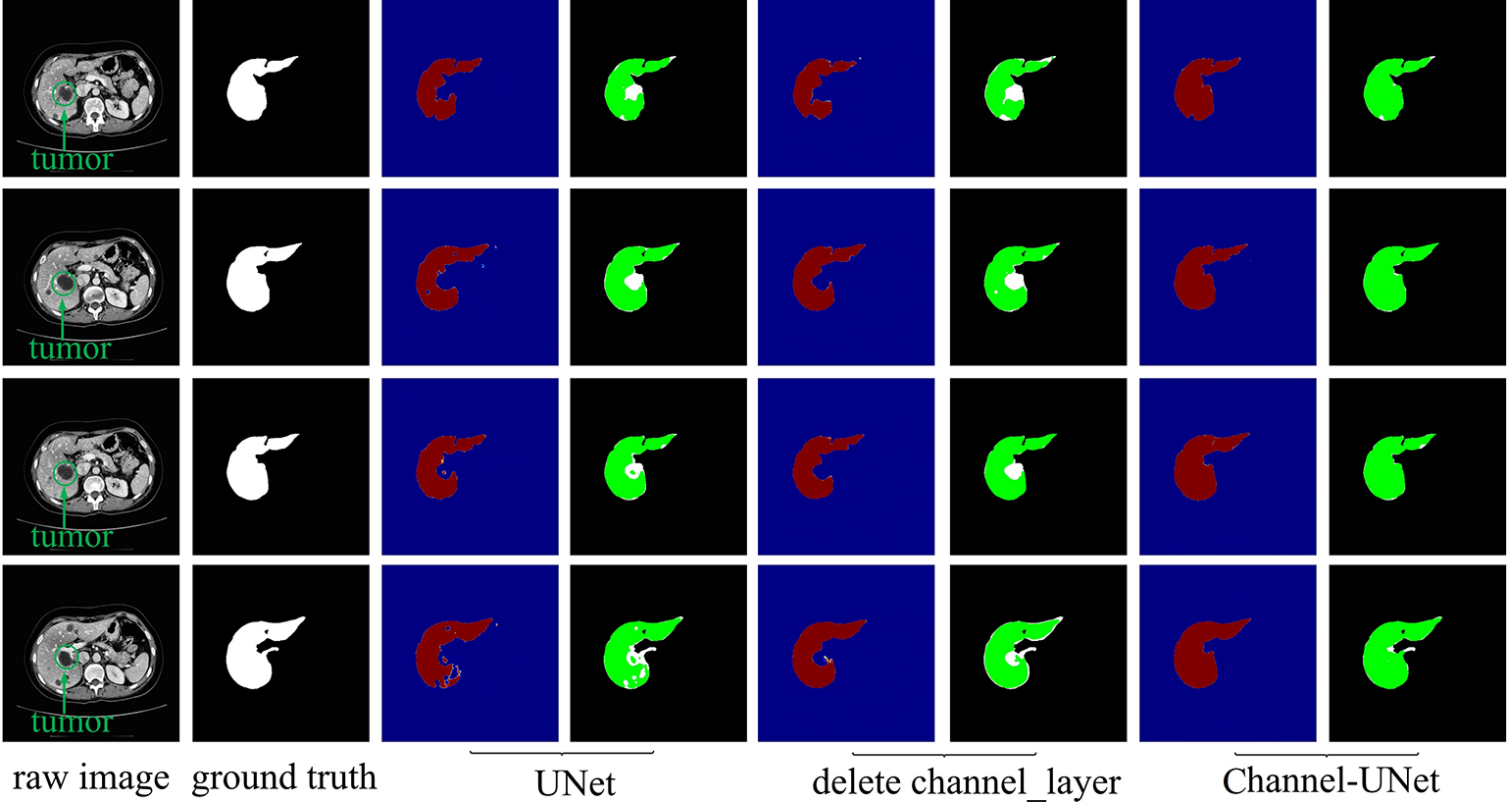
Liver segmentation results by ablation study on validation dataset. The red part is the heat map of the output, and the green part represents the segmentation of the liver.

**Figure 9:**
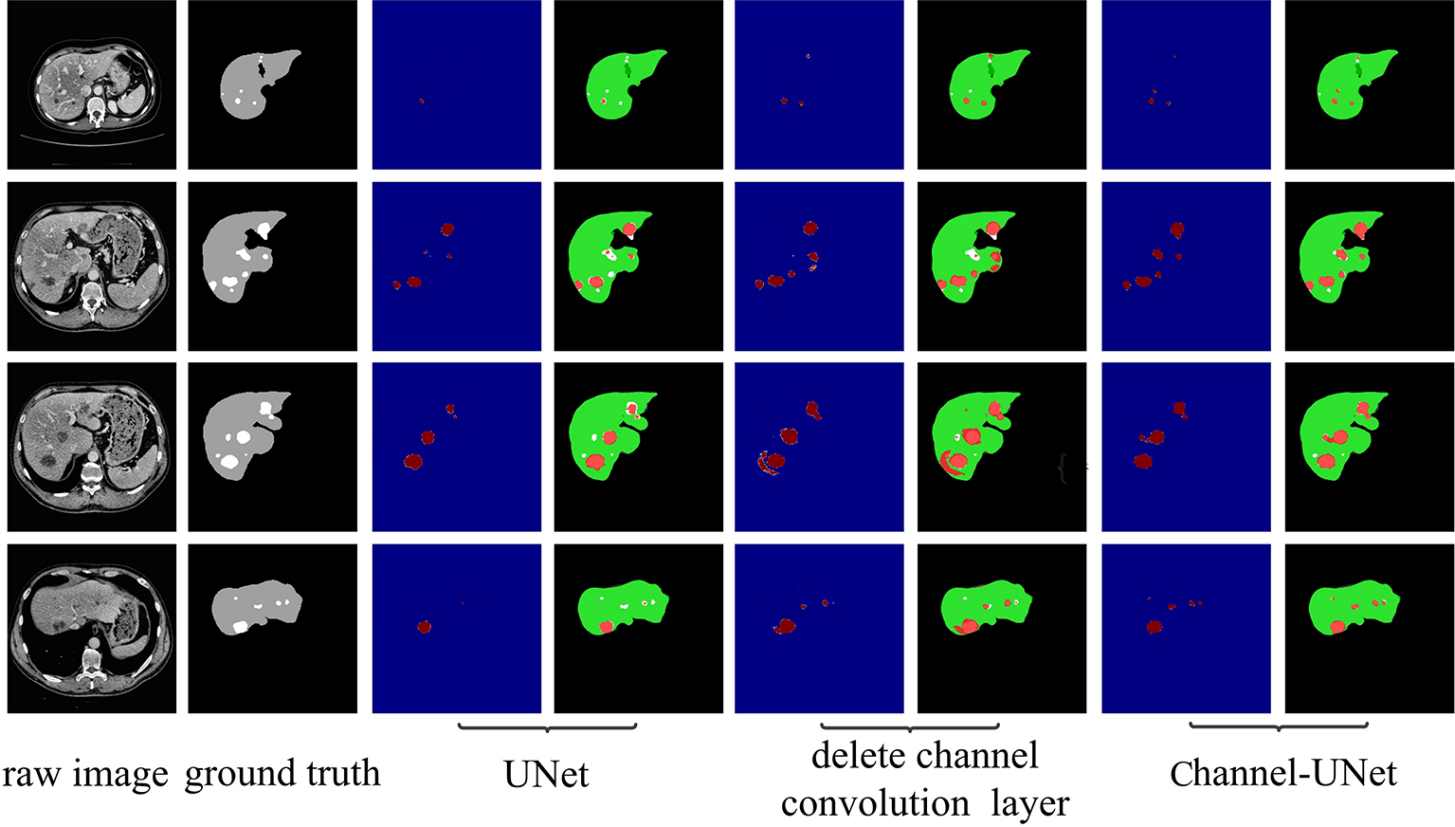
Tumor segmentation results by ablation study on validation dataset. The gray regions denote the true liver while the white ones denote the true tumors. Columns 3, 5, and 7 show the heat map of tumor segmentation. Columns 4, 6, and 8 show the results of tumor segmentation, where red represents the tumor and green represents the liver.

To verify the validity of the spatial channel-wise convolution layer structure, we compared the segmentation effects of removing the spatial channel-wise convolution layer with Channel-UNet on liver and tumor. Compared with the network after removing the spatial channel-wise convolution layer, the Channel-UNet proposed in this paper achieves good segmentation results in two measurement indicators of liver and tumors segmentation, as shown in **Table 2**. The improvement of segmentation accuracy indicates that the information between the pixels on (*x*, *y*)-plane extracted by spatial channel-wise convolution is helpful to the recognition of tumors and liver, especially when the pixel values of tumors and background are close. **Figures 8** and **9** show the segmentation results of removing spatial channel-wise convolution layer and Channel-UNet on the 3Dircadb dataset. The results show that the segmentation effect is better after adding spatial channel-wise convolution and our method can resolve the problems of over-segmentation and under segmentation.

**Table 2:** Segmentation results by ablation study of our method on the test dataset.

Method	Dice	
	Tumor	Liver
UNet	91.2	94.3
UNet+conv	89.5	92.4
UNet+conv+channelconv (Channel-UNet)	94.7	98.4

conv, convolutional layer; channelconv, spatial channel-wise convolution layer.

### Comparison With Other Methods

To verify the advantage of our method, we conduct comparison experiments on 3Dircadb dataset, which is publicly available and offers a higher variety and complexity of livers and tumors. On the 3Dircadb dataset, we compare the cross validation of Channel-UNet with the current best method (Li et al., 2018b), as well as other methods. The liver and tumors segmentation accuracy is shown in **Tables 3** and **4**. Compared with the current best method (Li et al., 2018b), the Dice values of liver and tumors segmentation by Channel-UNet are 0.984 and 0.940 (as shown in **Tables 3** and **4**), which is slightly superior to it. Besides, for (Li et al., 2018b), the number of parameters is 61.4 × 10^6^ and the training time is *9* h, while for Channel-UNet, the number of parameters is 45.6 × 10^6^ and the training time is *6* h. Compared with (Li et al., 2018b), the number of parameters of our method reduces by 25.7% and the training time of our method reduces by 33.3%. Our method optimizes the mapping relationship of spatial information between pixels extracted by spatial channel-wise convolution, and reduces the computational complexity and training parameters at the same time.

**Table 3:** Comparison of liver segmentation results.

Model	VOE (%)	RVD (%)	ASD (mm)	RMSD (mm)	DICE (%)
UNet (Chlebus et al., 2017)	14.21 ± 5.71	–0.05 ± 0.10	4.33 ± 3.39	8.35 ± 7.54	0.923 ± 0.03
ResNet (Han, 2017)	11.65 ± 4.06	–0.03 ± 0.06	3.91 ± 3.95	8.11 ± 9.68	0.938 ± 0.02
Christ (Christ et al., 2016)	10.7	−1.4	1.5	24.0	0.943
X. Li et al. (Li et al., 2018b)	10.20 ± 3.44	−0.01 ± 0.05	4.06 ± 3.85	9.63 ± 10.41	0.947 ± 0.01
H. Jiang et al. (Jiang et al., 2019)	–	–	–	–	0.945 ± 0.02
**Ours**	**9.52 ± 4.65**	−**0.02 ± 0.07**	**8.43 ± 9.39**	**14.21 ± 5.71**	**0.946 ± 0.03**
Li et al. + (Li et al., 2015)	9.15 ± 1.44	−0.07 ± 3.63	1.55 ± 0.39	3.15 ± 0.98	–
Moghbel + (Moghbel et al., 2016)	5.95	7.49	–	–	0.911
Lu et al. + (Lu et al., 2017)	9.36 ± 3.34	0.97 ± 3.26	1.89 ± 1.08	4.15 ± 3.16	–
X. Li et al. + (Li et al., 2018b)	3.57 ± 1.66	0.01 ± 0.02	1.28 ± 2.02	3.58 ± 6.58	0.982 ± 0.01
**Ours +**	**4.02 ± 1.42**	−**0.02 ± 0.03**	**1.24 ± 1.02**	**3.48 ± 4.16**	**0.984 ± 0.01**

+ denotes the method using augmented training dataset; - denotes the unreported result.

**Table 4:** Comparison of tumors segmentation result.

Model	VOE (%)	RVD (%)	ASD (mm)	RMSD (mm)	DICE (%)
UNet (Chlebus et al., 2017)	62.55 ± 22.36	0.380 ± 1.95	11.11 ± 12.02	16.71 ± 13.81	0.51 ± 0.25
Christ (Christ et al., 2016)	–	–	–	–	0.56 ± 0.26
ResNet (Han, 2017)	56.47 ± 13.62	−0.41 ± 0.21	6.36 ± 3.77	11.69 ± 7.60	0.60 ± 0.12
X. Li et al. (Li et al., 2018b)	49.72 ± 5.20	−0.33 ± 0.10	5.29 ± 6.15	11.11 ± 29.14	0.65 ± 0.02
H. Jiang et al. (Jiang et al., 2019)	1.354	0.129	1.074	1.412	0.62 ± 0.07
**Ours**	**41.54 ± 4.32**	**0.159 ± 5.03**	**2.04 ± 4.32**	**2.12 ± 5.52**	**0.66 ± 0.03**
Foruzan * (Foruzan and Chen, 2016)	30.61 ± 10.44	15.97 ± 12.04	4.18 ± 9.60	5.09 ± 10.71	0.82 ± 0.07
Wu et al. * (Wu et al., 2017)	29.04 ± 8.16	−2.20 ± 15.88	0.72 ± 0.33	1.10 ± 0.49	0.83 ± 0.06
Li et al. + (Li et al., 2015)	14.4 ± 5.3	−8.10 ± 2.1	2.4 ± 0.8	2.9 ± 0.7	–
Moghbel + (Moghbel et al., 2016)	22.78 ± 12.15	8.59 ± 18.78	–	–	0.75 ± 0.15
Sun et al. + (Sun et al., 2017)	15.6 ± 4.3	5.80 ± 3.5	2.0 ± 0.9	2.90 ± 1.5	–
X. Li et al. + (Li et al., 2018b)	11.68 ± 4.33	−0.01 ± 0.05	0.58 ± 0.43	1.87 ± 2.33	0.937 ± 0.02
**Ours +**	**13.68 ± 3.71**	**2.01 ± 0.05**	**0.46 ± 0.43**	**1.67 ± 2.33**	**0.940 ± 0.02**

* denotes the semi-automatic method; + denotes the method using augmented training dataset; - denotes the unreported result.

## Discussion

Automated segmentation of liver and tumor plays an important role in clinical diagnosis and treatment planning. In this paper, we first propose spatial channel-wise convolution to extract the mapping relationship of spatial information between pixels by spatial channel-wise convolution. Essentially, spatial channel-wise convolution is also a convolutional operation. The difference between traditional convolution and spatial channel-wise convolution is that the convolutional kernel slides along the channel direction, while the spatial channel-wise convolution slides perpendicularly to the plane direction of the channel. Compared with the image segmentation algorithm which only adds multi-scale image information(Lin et al., 2017; Peng et al., 2017), spatial channel-wise convolution can extract the mapping relationship of spatial information between the pixels in the convolution network, so that multi-scale information can be filtered through spatial information extracted by spatial channel-wise convolution when adding multi-scale information. What is more, compared with the image segmentation algorithm which optimizes the extracted information (Hong et al., 2016), we do not use attention mechanism that spatial channel-wise convolution layer obtains probability graph model through soft-max activation function, and then multiplies it with feature graph. On the contrary, we adopt the method of concatenating spatial channel-wise convolution layer and convolution layer directly along the channel direction. This is because spatial channel-wise convolution is not the optimization of convolution layer information, but the extraction of mapping relationship of spatial information between pixels of convolution layers. This information exists in convolution layer but cannot be learned by parameters. Therefore, concatenating operation can retain the mapping relationship of spatial information extracted by spatial channel-wise convolution to the maximum extent.

Secondly, we add convolution layer before spatial channel-wise convolution to increase the receptive field of spatial channel-wise convolution. In this way, the spatial channel-wise convolution can extract the mapping relationship of spatial information on different feature maps. The experimental results show that the dice value of liver segmentation increases first and then decreases with the increase of spatial channel-wise convolution. It shows that too much increase of the visual field of spatial channel-wise convolution cannot improve the segmentation effect. Therefore, we did not use the dilated convolution (Yu and Koltun, 2015), which can greatly expand the receptive field of spatial channel-wise convolution.

Finally, we propose an end-to-end network, called Channel-UNet, for automatic segmentation of liver and tumors. We find out that it can solve the problems of over-segmentation and under segmentation in image segmentation. We compare our method with current best method (Li et al., 2018b), the Dice value of our method is slightly superior to (Li et al., 2018b), and number of parameters of our method is reduced by 25.7% and the training time is reduced by 33.3%. This is definitely important in clinical practice, especially when there is a lot of slice data to be processed. Our method can provide more efficient and accurate liver and tumors segmentation for clinical analysis.

Additionally, in order to better test the performance of the Channel-UNet under different segmentation drawbacks, we test the performance of the network for liver segmentation under different segmentation drawbacks according to the segmentation drawbacks given by 3Dircadb. **Table 5** shows 10 segmentation drawbacks and the serial number of validation data, which is the original patient index of the 3Dircadb dataset. As shown in **Table 5**, our method improves the Dice by 1.01–3.91% compared with the baseline under different segmentation drawbacks, and Dice of liver increases by 2.31% on average in the case of segmentation drawbacks compared with 1.01% in the case of no segmentation drawbacks. Based on the comparison results, it can be concluded that the improvement of segmentation accuracy is mainly attributed to the improvement of liver with segmentation drawbacks. This is mainly because the spatial channel-wise convolution layer extracts the mapping relationship of spatial information between pixels, which makes the original blurred boundary become conducive to segmentation. Also, it can be found in **Table 5** that the improvement of segmentation is limited when the network encounters the drawbacks of heart and diaphragm, as these interference organs usually occur in fewer slices. However, the proposed method is a supervised learning method. When segmenting the liver and tumors, we need to train the Channel-UNet with the corresponding liver and tumor dataset, which make the proposed method heavily dependent on the scale and quality of the dataset. In future work, we plan to integrate the Channel-UNet with transfer learning (Van Opbroek et al., 2018) and small sample learning to address the above challenges. Besides, researchers have shown that using convolution kernels of different sizes can fuse information of different scales and improve classification accuracy (Szegedy et al., 2015), we plan to investigate the spatial channel-wise convolution kernels with different sizes to extract spatial mapping relations at different scales and improve the segmentation accuracy of liver tumors.

**Table 5:** Effectiveness of our method regarding to the segmentation drawbacks.

Segmentation drawbacks	Test number	Dice (%)	
		Baseline	Channel-UNet
Stomach	20	94.25	97.65 (+3.40)
Pancreas	15	95.55	97.62 (+2.07)
Duodenum	19	95.35	97.42 (+2.07)
Metal	3	96.90	98.15 (+1.25)
Heart	4	96.74	97.97 (+1.23)
Diaphragm	5	96.54	97.76 (+1.22)
Spleen	7	90.82	94.43 (+3.91)
Colon	9	96.65	98.64 (+1.99)
Muscles	16	93.16	96.07 (+2.91)
Esophagus	18	93.77	96.81 (+3.40)
No	12	97.85	98.86 (+1.01)

Baseline is the UNet with data augmentation.

## Conclusion

In this paper, we propose the spatial channel-wise convolution layer to extract mapping relationship of spatial information between pixels, raise the iterative extending learning strategy that extends the receptive field of single spatial channel-wise convolution layer by layer and optimizes the mapping relationship of spatial information by back propagation, and finally design an end-to-end network named Channel-UNet to solve the problems of over-segmentation and under-segmentation. The proposed Channel-UNet achieves superior Dice value of liver and tumors segmentation, and significantly reduces the number of parameters and training time compared with state-of-the-arts.

## Data Availability Statement

The datasets generated for this study are available on request to the corresponding author.

## Author Contributions

YC, KW, XL, ZY carried out the conception design and experiment, and organized and revised the manuscript. YQ, QW, and P-AH participated in the experimental results analysis and discussion. All authors read and approved the manuscript.

## Funding

The work is supported by grants from National Natural Science Foundation of China (No. U1813204, No. 61902386), Shenzhen Science and Technology Program (No.JCYJ20160429190300857, No. JCYJ20170413162617606), HK RGC TRS project T42-409/18-R, the CUHK T Stone Robotics Institute, National Natural Science Foundation of China (No. 61802386) and Natural Science Foundation of Guangdong (No.2018A030313100).

## Conflict of Interest

The authors declare that the research was conducted in the absence of any commercial or financial relationships that could be construed as a potential conflict of interest.

## Footnotes

1
https://www.ircad.fr/research/3dircadb/
